# Tests for Impurities in Water Available for Physicians’ Use

**Published:** 1886-10-20

**Authors:** Curtis C. Howard

**Affiliations:** Professor of Chemistry, Starling Medical College, Columbus, Ohio


### TESTS FOR IMPURITIES IN WATER AVAILABLE FOR PHYSICIANS' USE.

BY CURTIS C. HOWARD, M. C.,

**Professor of Chemistry, Starling Medical College, Columbus, Ohio.**

The most valuable tests for determining the organic impurity in drinking water, as a rule, require an amount of apparatus and practice that places them beyond the reach of the practising physician. The estimation of organic carbon and nitrogen, of free and albuminoid ammonia, and of nitrogen as nitrates and nitrites are illustrations of this fact.

A method that requires no apparatus and but one or two reagents, and the results of which, manifested either by the appearance of a color or a precipitate, are at once recognized by the eye; such a method will be found of value when circumstances prevent a more complete analysis.

In an examination of a considerable number of samples of well water, my attention has been drawn to the significance attaching to the presence of the nitrites and chlorides.

The nitrogen of organic bodies is converted in the process of decay into ammonia, and exposed to oxidizing agencies is oxidized to nitrous and nitric acids, which combine with bases to form nitrites and nitrates. The former are of special interest to us, and I believe that a water organically pure should not contain more than one thousandth part per hundred thousand of nitrous acid, and that the presence of three or four times this quantity is sufficient to condemn a water.

A number of substances have been used as tests for these nitrites. The most delicate reagents, and those I have found to act most satisfactorily, are sulphuric acid and naphthylamine hydrochloride. If water containing not more than one thousandth part per hundred thousand of nitrous acid be treated with a drop of hydrochloric acid and a drop each of solutions of these reagents, after standing ten or fifteen mi utes, only the faintest tint of pink will be perceived. If a marked pink be produced, the quantity is sufficient to condemn a water.

A number of substances have been used as tests for these nitrites. The most delicate reagents, and those I have found to act most satisfactorily, are sulphuric acid and naphthylamine hydrochloride. If water containing not more than one thousandth part per hundred thousandth of nitrous acid be treated with a drop of hydrochloric acid and a drop each of solutions of these reagents, after standing ten or fifteen minutes, only the faintest tint of pink will be perceived. If a marked pink be produced, the quantity of nitrites is sufficient to indicate serious contamination. In sewage and in the water from a few wells, the color was of a deep carmine and the quantity present twenty to sixty-six times the limit stated.

Another constituent of importance is chlorine combined with sodium, as sodium chloride or common salt. Since this is found in the fluids of the body, and urine contains five hundred parts per hundred thousaud of chlorine, mixtures of animal excreta with water will increase the quantity of chlorine found therein. Two or three wells have been found in Columbus containing less than two parts per hundred thousand of chlorine, but the majority contain from five to twenty parts per hundred thousand, while one well in an adjoining town contained more than fifty parts.

The reagents for chlorides are nitric acid and silver nitrate, which produce in water containing chlorides a white precipitate of silver chloride. In water containing one or two parts of chlorine per hundred thousand, the precipitate is so slight that it appears as an opalescence, while with ten or twenty parts a precipitate is produced. The appearance of a marked precipitate indicates the presence of a sufficient quantity of chlorides to justify the rejection of the water. A few quantitative results are added.

| | Parts per 100 000. | |
|---|---|---|
| | Nitrous Acid. | Chlorine. |
| Maximum thirty-four samples | .020 | 51.11 |
| Minimum thirty-four samples less than | .001 | 1.83 |
| Average eighteen samples | .0064 | 15.20 |
| Average sixteen samples | .0011 | 3.82 |
| City water, October 30th, 1885 | trace | .74 |
| Sewage, April 9th, 1885 | .0659 | 6.80 |

—*The Sanitarian.*

